# High-*b*-value spherical tensor encoding diffusion-weighted imaging of acute stroke

**DOI:** 10.1007/s11604-026-01960-4

**Published:** 2026-02-17

**Authors:** Takashi Yoshiura, Kiyohisa Kamimura, Yushi Nagano

**Affiliations:** 1 Department of Radiology, Graduate School of Medical and Dental Sciences, Kagoshima University, 8-35-1 Sakuragaoka, Kagoshima, 890-8544 Japan; 2 Department of Advanced Radiological Imaging, Graduate School of Medical and Dental Sciences, Kagoshima University, Kagoshima, Japan; 3 Department of Neurosurgery, Graduate School of Medical and Dental Sciences, Kagoshima University, Kagoshima, Japan

In brain diffusion-weighted imaging (DWI), the b-value is typically set at 1000 s/mm^2^, but early ischemic lesions may appear only as subtle hyperintensities. Higher b-values can improve lesion-to-normal contrast, although high–b-value DWI shows intrinsic hyperintensity in the normal white matter (WM) due to diffusion anisotropy, which may obscure lesions or mimic pathology [1]. Spherical tensor encoding (STE) DWI provides isotropic diffusion weighting in a single acquisition [2, 3]. Unlike conventional linear tensor encoding (LTE), STE DWI is insensitive to macroscopic diffusion anisotropy, thereby suppressing WM hyperintensity at high b-values.

A 75-year-old man with acute embolic infarction treated with intravenous alteplase and mechanical thrombectomy underwent follow-up MRI two days after the symptom onset. DWI included LTE at b = 1000 s/mm^2^ (LTEb1000), LTE at b = 3000 s/mm^2^ (LTEb3000), and STE at b = 3000 s/mm^2^ (STEb3000) (Fig. 1, see Supplementary Table for imaging parameters). Although LTEb3000 increased contrast between ischemic lesions and the adjacent cerebral cortex, WM hyperintensity obscured lesion boundaries and major WM tracts appeared as focal hyperintensities, potentially mimicking ischemic lesions. In contrast, STEb3000 provided superior lesion-to-normal tissue contrast without WM hyperintensity interference.

**Fig. 1:**
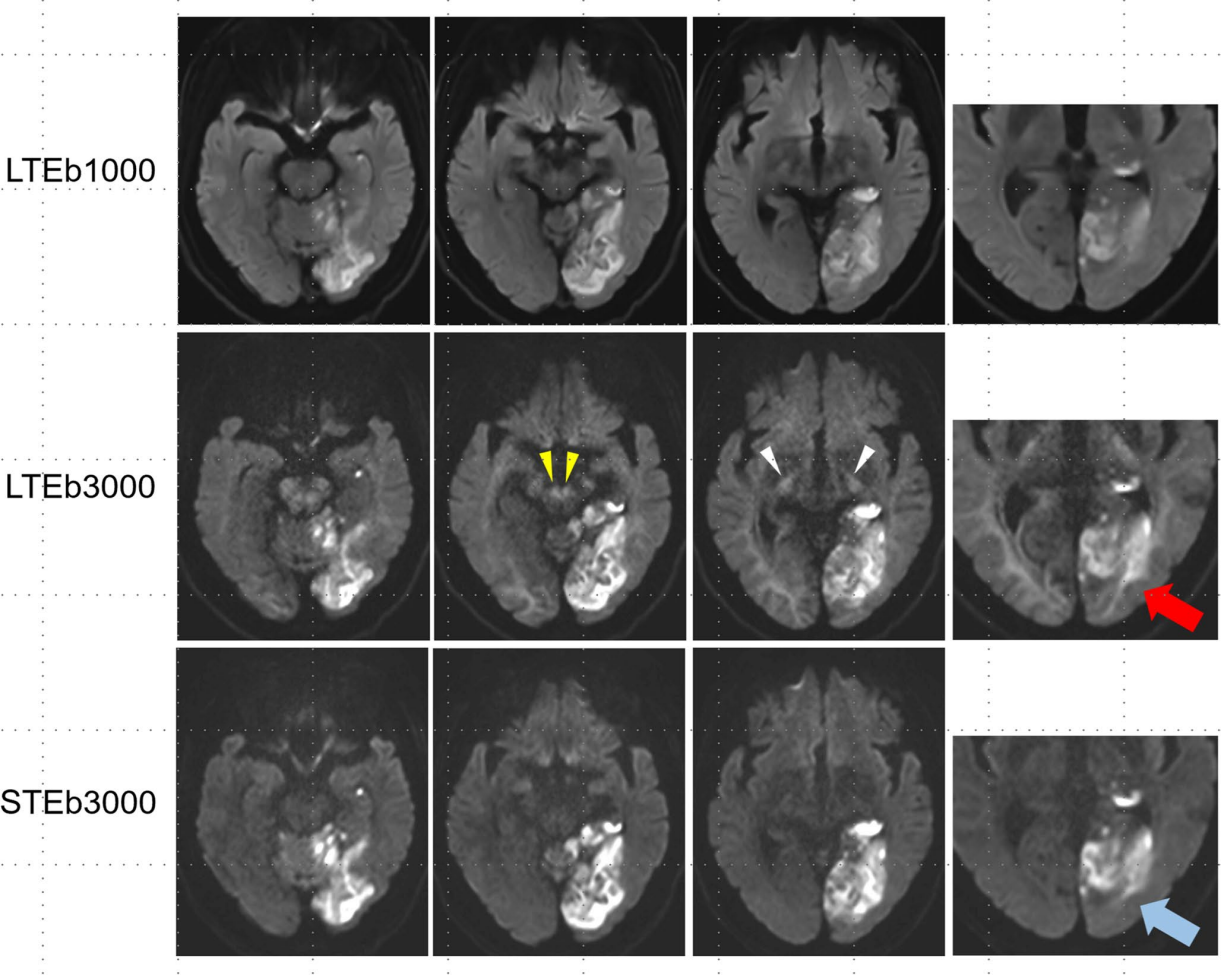
Compared with LTEb1000, LTEb3000 provided increased lesion-to-normal tissue contrast; however, intrinsic WM hyperintensity obscured the ischemic lesion boundary (red arrow). Focal hyperintensities in the prominent WM tracts, such as the superior cerebellar peduncles (yellow arrowheads) and the internal capsule (white arrowheads) could be mistaken for ischemic lesions. STEb3000 effectively suppressed anisotropic WM signals, producing greater lesion-to-normal contrast and clearer lesion delineation (blue arrow)

## Supplementary Information

Below is the link to the electronic supplementary material.

Supplementary Material 1
