# Supplementary material for: High-b-value spherical tensor encoding diffusion-weighted imaging of acute stroke
Source: Jpn J Radiol. 2026 Feb 17;44(6):1136–8. doi: 10.1007/s11604-026-01960-4 (PMC13222185; doi:10.1007/s11604-026-01960-4)
Supplement: Supplementary file 1 — Supplementary Material 1 [file 11604_2026_1960_MOESM1_ESM.pdf]

Imaging parameters for diffusion-weighted imaging

	LTE b1000	LTE b3000	STE b3000
Sequence type	Single-shot echo-planar		
b values (s/mm2)	0, 1000	0, 3000	0, 3000
Repetition time (ms)	4000	4000	4000
Echo time (ms)	80	113	113
Flip angle	90	90	180
Number of averages	2	3	10
Filed-of-view (mm)	230	230	230
Matrix	256	256	220
Slice thickness (mm)	5	5	5
Inter-slice gap (mm)	1	1	1
Acquisition time (s)	47	58	55

LTEb1000, linear tensor encoding at a b value of 1000 s/mm^2; LTEb3000, linear tensor encoding at a b value of 3000 s/mm^2; STEb3000, spherical tensor encoding at a b value of 3000 s/mm^2
